# Measurement and Data Transmission Validity of a Multi-Biosensor System for Real-Time Remote Exercise Monitoring Among Cardiac Patients

**DOI:** 10.2196/rehab.3633

**Published:** 2015-03-20

**Authors:** Jonathan C Rawstorn, Nicholas Gant, Ian Warren, Robert Neil Doughty, Nigel Lever, Katrina K Poppe, Ralph Maddison

**Affiliations:** ^1^National Institute for Health InnovationUniversity of AucklandAucklandNew Zealand; ^2^Department of Sport and Exercise ScienceUniversity of AucklandAucklandNew Zealand; ^3^Department of Computer ScienceUniversity of AucklandAucklandNew Zealand; ^4^Department of MedicineUniversity of AucklandAucklandNew Zealand

**Keywords:** telemedicine, remote sensing technology, telemetry, smartphone, mHealth, rehabilitation, cardiac rehabilitation

## Abstract

**Background:**

Remote telemonitoring holds great potential to augment management of patients with coronary heart disease (CHD) and atrial fibrillation (AF) by enabling regular physiological monitoring during physical activity. Remote physiological monitoring may improve home and community exercise-based cardiac rehabilitation (exCR) programs and could improve assessment of the impact and management of pharmacological interventions for heart rate control in individuals with AF.

**Objective:**

Our aim was to evaluate the measurement validity and data transmission reliability of a remote telemonitoring system comprising a wireless multi-parameter physiological sensor, custom mobile app, and middleware platform, among individuals in sinus rhythm and AF.

**Methods:**

Participants in sinus rhythm and with AF undertook simulated daily activities, low, moderate, and/or high intensity exercise. Remote monitoring system heart rate and respiratory rate were compared to reference measures (12-lead ECG and indirect calorimeter). Wireless data transmission loss was calculated between the sensor, mobile app, and remote Internet server.

**Results:**

Median heart rate (-0.30 to 1.10 b∙min^-1^) and respiratory rate (-1.25 to 0.39 br∙min^-1^) measurement biases were small, yet statistically significant (all *P*≤.003) due to the large number of observations. Measurement reliability was generally excellent (rho=.87-.97, all *P*<.001; intraclass correlation coefficient [ICC]=.94-.98, all *P*<.001; coefficient of variation [CV]=2.24-7.94%), although respiratory rate measurement reliability was poor among AF participants (rho=.43, *P*<.001; ICC=.55, *P*<.001; CV=16.61%). Data loss was minimal (<5%) when all system components were active; however, instability of the network hosting the remote data capture server resulted in data loss at the remote Internet server during some trials.

**Conclusions:**

System validity was sufficient for remote monitoring of heart and respiratory rates across a range of exercise intensities. Remote exercise monitoring has potential to augment current exCR and heart rate control management approaches by enabling the provision of individually tailored care to individuals outside traditional clinical environments.

## Introduction

Cardiovascular diseases remain the leading cause of morbidity and mortality worldwide, accounting for around one third (approximately 17 million) of deaths globally, with the greatest proportion of deaths attributed to coronary heart disease (CHD) [1]. Cardiac rehabilitation (CR) is an essential component of CHD management [2,3], and international guidelines consistently identify exercise training as a central element of CR [4-6]. The beneficial effects of exercise-based CR (exCR) on all-cause and cardiac mortality are comparable with comprehensive CR [7-10], and exercise training can concurrently improve an array of modifiable cardiac risk factors including hypertension, dyslipidemia, insulin resistance, overweight and obesity, and exercise capacity [10-15]. Despite these benefits, many eligible patients are not referred for CR [16], and uptake is low among those who are referred [17,18]. Common participation barriers include transport limitations, work commitments, and inconvenient program scheduling [19]. Among those who do undertake CR, adherence to prescribed exercise is poor with up to 50% of participants dropping out of regular exercise within 6 months of program completion [20-22]. It is clear traditional CR delivery models do not meet the needs of many eligible patients, and innovation is required to enhance participation and adherence.

Home-based exCR has been introduced to broaden access and participation and confers similar improvements in mortality, cardiac events, and cardiac risk factors compared to center-based CR [23]. Home-based programs overcome several traditional participation barriers, but many do not include physiological monitoring that is typical during center-based exCR. In addition to concerns about patient safety, a lack of physiological monitoring also restricts the potential to individualize and optimally manage exercise prescription. As the beneficial effects of exCR are dose-dependent [11,24,25], remote physiological monitoring may help home-based exCR participants to achieve recommended exercise training loads and improve program outcomes.

Physiological monitoring has recently been identified as a particularly important direction for the future development of home-based exCR [26], but to date, most telehealth CR interventions have utilized fixed-line communication tools (eg, telephone, Internet, videoconferencing, transtelephonic electrocardiogram [ECG]) that constrain participants within the home environment. Recent advances in mobile sensor technologies and rapidly growing access to mobile broadband [27] enable real-time remote physiological monitoring outside fixed-line communication networks, and these technologies should be integrated into telehealth CR [28].

A survey of wearable physiological monitoring devices identified several key requirements including measurement validity, data transmission integrity, real-time data processing, ease of use, and scalability [29]. While few existing monitoring systems addressed all requirements the commercially available BioHarness (Zephyr Technology) scored highly [29]. This multi-parameter wireless biosensor quantifies heart rate, single lead ECG, respiratory rate, tri-axial body acceleration, and torso posture via sensors embedded in a textile chest strap or compression-fit vest. On-board memory and Bluetooth connectivity enable data to be stored locally or transmitted wirelessly to compatible devices such as smartphones, tablets, and computers. The low-profile design, ease of use, and advanced array of sensors make this device well suited for remote exercise monitoring. Early model BioHarness devices have been validated [30-35]; however, the current model has yet to be evaluated in either clinical or non-clinical populations.

Most wearable physiological sensors do not support long-range data transmission to remotely located monitoring stations. Therefore, remote monitoring requires physiological sensors to be combined with devices capable of collating and transmitting sensor data to remote monitoring stations for review and action by health care professionals. Smartphones are a preferable intermediary as, in combination with appropriate mobile or Web apps, they provide a ready-to-use mobile platform capable of logging and transmitting data via ubiquitous wireless data networks (eg, Bluetooth, Wi-Fi, 3G, and 4G). Portability, compatibility with several data networks, substantial computational capability, and routine integration of motion and location sensors further enhance the potential utility of smartphones for remote exercise monitoring. Moreover, continued rapid global smartphone market penetration growth [36] will likely reduce the necessity for health care providers to supply smartphones to would-be remote monitoring system end users. To date, there is a lack of published research combining physiological sensors with mobile data transmission technologies. A system comprising ECG and global positioning system (GPS) sensors, and a smartphone has been evaluated for remotely monitoring cardiac patients during exercise [37]. Remote data transmission was interrupted during 8.6% of completed exercise sessions; however, the amount of data lost and the subsequent impact on real-time remote monitoring were not described.

We have developed a custom mobile app and middleware platform to provide real-time transmission of physiological and clinical data, via smartphones, to remotely located monitoring centers [38]. Bi-directional communication capability enables health care professionals to provide users with instantaneous feedback that could prompt rapid changes in exercise behavior, enhance exercise self-efficacy, deliver educational information and provide support. Frequent access to remotely recorded heart rate data during rest, activities of daily living, and exercise could enable physicians to assess the impact of pharmacological intervention on heart rate control. In combination with the communication capability, this could assist physicians to titrate AF patients’ medications in order to achieve optimal heart rate control. Our platform has shown promise in preliminary proof of concept research; however, a robust assessment of wireless data transmission reliability is required.

This study aimed to evaluate the sensor measurement validity and wireless data transmission reliability of a remote physiological monitoring system comprising the BioHarness, custom app, and middleware platform among individuals in sinus rhythm. Given that AF is the most common sustained cardiac arrhythmia and is a common comorbidity in CHD [39], system validity was also assessed in individuals with AF to determine whether the sensor was robust to a common cardiac dysrhythmia.

## Methods

### Overview

A dual-phase cross-sectional study was conducted to assess system validity among convenience samples of healthy recreationally active individuals in sinus rhythm (ie, systole initiated at the sinoatrial node and proliferated via normal cardiac conduction pathways; Phase One), and individuals with AF (Phase Two). Phase One participants were recruited via contacts and local sport clubs. Phase Two participants were recruited via outpatient cardiology clinics. This dual-phase approach enabled safe assessment of sensor measurement validity across a broad range of exercise intensities. Phase One participants completed constant, intermittent, and incremental intensity exercise at moderate to maximal levels of intensity. Phase Two participants completed constant intensity exercise and simulated daily activities at low to moderate levels of intensity. Phase One was approved by the University of Auckland Human Participants Ethics Committee (2011/7674). Phase Two was approved by the New Zealand Health and Disability Ethics Committee (CEN/11/11058), respectively. All volunteers provided written informed consent. Procedures common to Phases One and Two are outlined below, followed by phase-specific exercise procedures.

### Common Procedures

**Figure 1:**
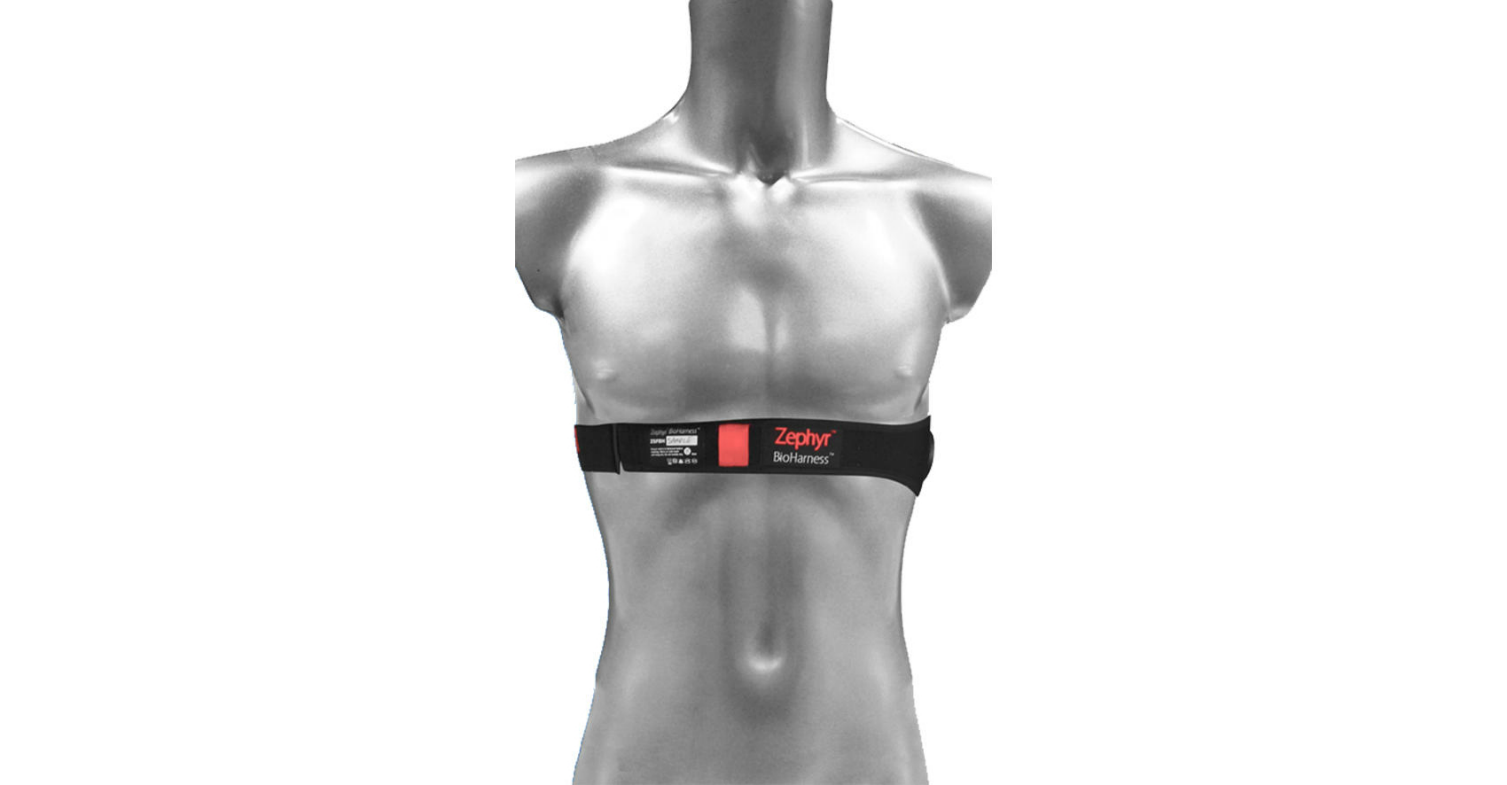
Zephyr BioHarness.

**Figure 2:**
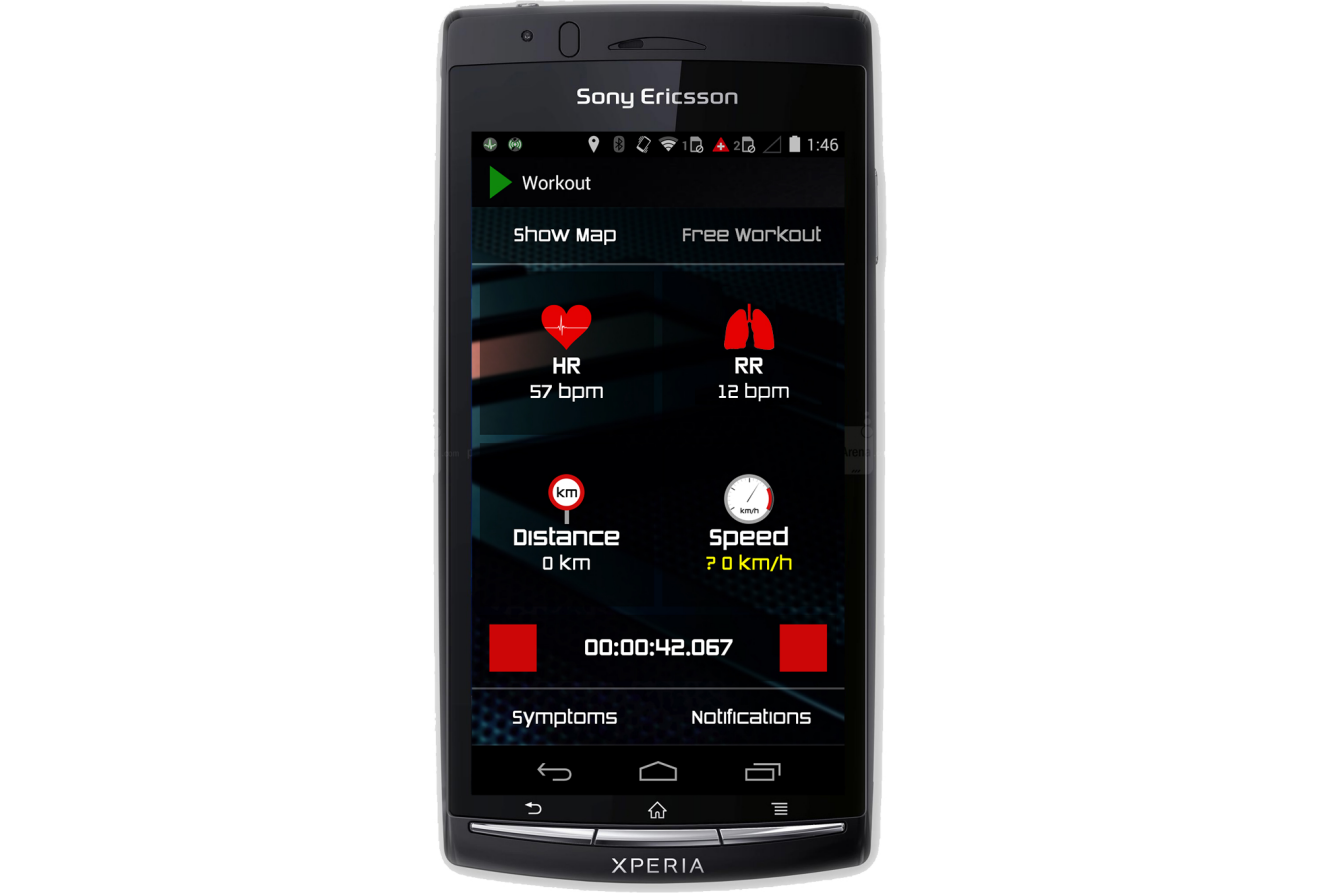
Custom mobile app screenshot.

The remote physiological monitoring system comprised the BioHarness (version 3 with chest strap; Figure 1), a smartphone (Xperia Arc S, Sony Ericsson Mobile Communications AB, Sweden) utilizing the Android operating system (v2.3.4, Google Inc.), a custom mobile app with integrated middleware platform (Figure 2), and a remote monitoring Internet server (Odin) [38]. Physiological data, transmitted to the smartphone via Bluetooth, were displayed throughout exercise, stored locally, and transmitted to the remote Internet server in near real-time (30-second data packet transmission interval).

On arrival at the laboratory, participants underwent baseline measurement of stature and body mass, and familiarization with exercise ergometers. Participants were instrumented with a 12-lead ECG (AT-110, Schiller AG), BioHarness, and indirect calorimeter (Metalyzer, Cortex Biophysik GmBH). Adhesive electrodes were applied at standard ECG sites following recommended skin preparation procedures [40], and electrical cables were secured to minimize signal artefact. Calorimeter gas sensors were calibrated via a two-point procedure using gases of known composition, the volume transducer was calibrated using a 3000 mLcalibration syringe (Hans Rudolph), and the internal barometer was calibrated against a mercury barometer (SK1256, Sato Keiryoki Manufacturing).

Activation of ECG, BioHarness, and calorimeter data logging followed a standardized procedure to ensure accurate data synchronization. Data were recorded during 180 seconds of seated rest prior to, and throughout exercise. A 60-second transition period was included prior to locomotive exercise to enable treadmill initiation.

### Phase One Exercise Procedures

During Phase One, participants completed three discrete bouts of treadmill running during two laboratory-based trials. During Trial One, participants ran on a motorized treadmill (EX200, Powersport) at 0% incline to determine the velocity eliciting 50% heart rate reserve (V_50%HRR_). Following instrumentation, participants completed an incremental protocol to assess peak oxygen uptake (V̇O_2_peak), operationally defined as the highest measured V̇O_2_. Treadmill velocity (V_50%HRR_) remained constant, and the incline was increased by 1% every 60 seconds until volitional exhaustion. Mean V̇O_2_ during the final 30 seconds of each workload was plotted as a function of treadmill incline, and inclines eliciting 50%, 66%, 70% and 90% V̇O_2_peak were derived via linear interpolation. After 30 minutes of rest, participants completed a 30 minute constant intensity treadmill running protocol (C30) at an incline eliciting 66% V̇O_2_peak. During Trial Two, participants completed a 30-minute intermittent intensity treadmill protocol (I30) comprising three repetitions of a 10-minute exercise block. Each exercise block included five sequential 2-minute stages at inclines eliciting 50%, 70%, 90%, 70%, and 50% V̇O_2_peak, respectively. Mean levels of exercise intensity were equivalent in the C30 and I30 protocols.

### Phase Two Exercise Procedures

During Phase Two, participants completed three bouts of exercise during a single laboratory-based trial. Participants self-selected light-to-moderate levels of exercise intensity during treadmill and cycle ergometer (Velotron, RacerMate Inc.) familiarization. Following instrumentation, participants undertook 10 minutes of treadmill walking, 10 minutes of cycling, and sequential 3-minute bouts of simulated daily activities (sweeping and vacuuming). Walk, cycle, and daily activity bouts were separated by 5 minutes of seated rest.

### Data Analysis

Reference heart rate measures were manually calculated from synchronized ECG waveforms as the average rate during the final 10 seconds of each minute. Reference respiratory rate was captured by the calorimeter at 0.10 Hz. BioHarness and calorimeter data were downloaded using the manufacturers’ software (BioHarness Log Downloader v1.0.24 and MetaSoft v3.9.3, respectively) and exported for manual analysis. BioHarness data were down-sampled to match reference measures. Data outside the manufacturers specified measurement ranges were excluded prior to analysis.

Phase One and Two data were analyzed separately following identical procedures using SPSS v20.0.0. Consistent with guidelines for assessing measurement validity in this field [41], a multi-faceted approach was undertaken to evaluate BioHarness heart rate and respiratory rate measurement accuracy and reliability. Heart rate and respiratory rate data were non-normally distributed, and a nonparametric analytical approach was implemented where necessary. Wilcoxon signed-rank tests for matched pairs were conducted to assess systematic biases between sensor and reference measures. Kruskal-Wallis analyses of variance were performed to assess the effect of Activity (Phase One: rest, transition, run; Phase Two: rest, transition, walk, cycle, sweep, vacuum) on measurement biases. Statistically significant main effects were explored using Dunn-Bonferroni corrected paired comparisons.

Spearman’s rank-order correlation coefficients (rho) and two-way random effects intraclass correlation coefficients (ICC) for absolute agreement were calculated to describe relative measurement reliability [41]. Absolute measurement reliability was assessed by calculating the standard error of measurement (SEM) and coefficient of variation (CV) [41] and a non-parametric approach to the 95% limits of agreement (LoA) similar to that described by Bland & Altman [42], in which the LoA were calculated as the 2.5^th^ and 97.5^th^ percentile ranked biases. The threshold for statistical significance was set at alpha<.05.

Wireless data transmission reliability was evaluated by determining data loss between the BioHarness, App, and remote monitoring server (Odin). Reference sample sizes were calculated as the product of exercise duration and sensor sampling frequency. These analyses utilized data logged at the BioHarness’ native summary frequency (1 Hz) as the aforementioned down-sampling procedures had potential to conceal intermittent data loss.

## Results

### Overview

Participant characteristics are summarized in Table 1. Ten and eight participants completed all Phase One and Two activity bouts, respectively. Unidentified trial-wide technical errors affected heart rate and respiratory rate measurements during two separate Phase Two trials. The outlying nature of these datasets was confirmed by 2 independent investigators, and they were excluded from analyses.

**Table 1:** Participant characteristics (Phase One: participants in sinus rhythm; Phase Two: participants with atrial fibrillation).

	Phase One, mean (SD)	Phase Two, mean (SD)
Sample size/male	10/6	8/5
Age, years	26.68 (3.26)	69.68 (9.53)
Body mass, kg	71.10 (11.53)	77.46 (18.81)
Stature, m	1.73 (0.06)	1.69 (0.12)
Peak oxygen consumption, ml∙kg^-1^∙min^-1^	50.82 (4.51)	Not assessed

### Measurement Accuracy

The BioHarness systematically underestimated heart rate (*z*=-3.01, *P*=.003) and respiratory rate (*z*=-21.57, *P*<.001) during Phase One, although the median biases were small (Tables 2 and 3). A statistically significant effect of Activity on measurement bias was detected for respiratory rate (H_2_=40.96, *P*<.001), but not heart rate (H_2_=0.83, *P*=.66). Dunn-Bonferroni corrected paired comparisons revealed systematic differences in respiratory rate measurement biases between all three levels of Activity (all *P*<.001 to *P*=.04; Table 3).

The BioHarness systematically overestimated heart rate (*z*=-3.28, *P*=.001) and respiratory rate (*z*=-4.47, *P*<.001) during Phase Two, although negative biases were observed during some activities (Tables 2 and 3). A statistically significant effect of Activity was detected on respiratory rate (H_5_=203.07, *P*<.001; Table 3), but not heart rate (H_5_=4.41, *P*=.49; Table 2). Dunn-Bonferroni-corrected paired comparisons revealed systematic differences in respiratory rate measurement biases between all levels of Activity (*P*<.001 to *P*=.02; Table 3) with the exception of walk and cycle (*P*=.12; Table 3).

**Figure 3:**
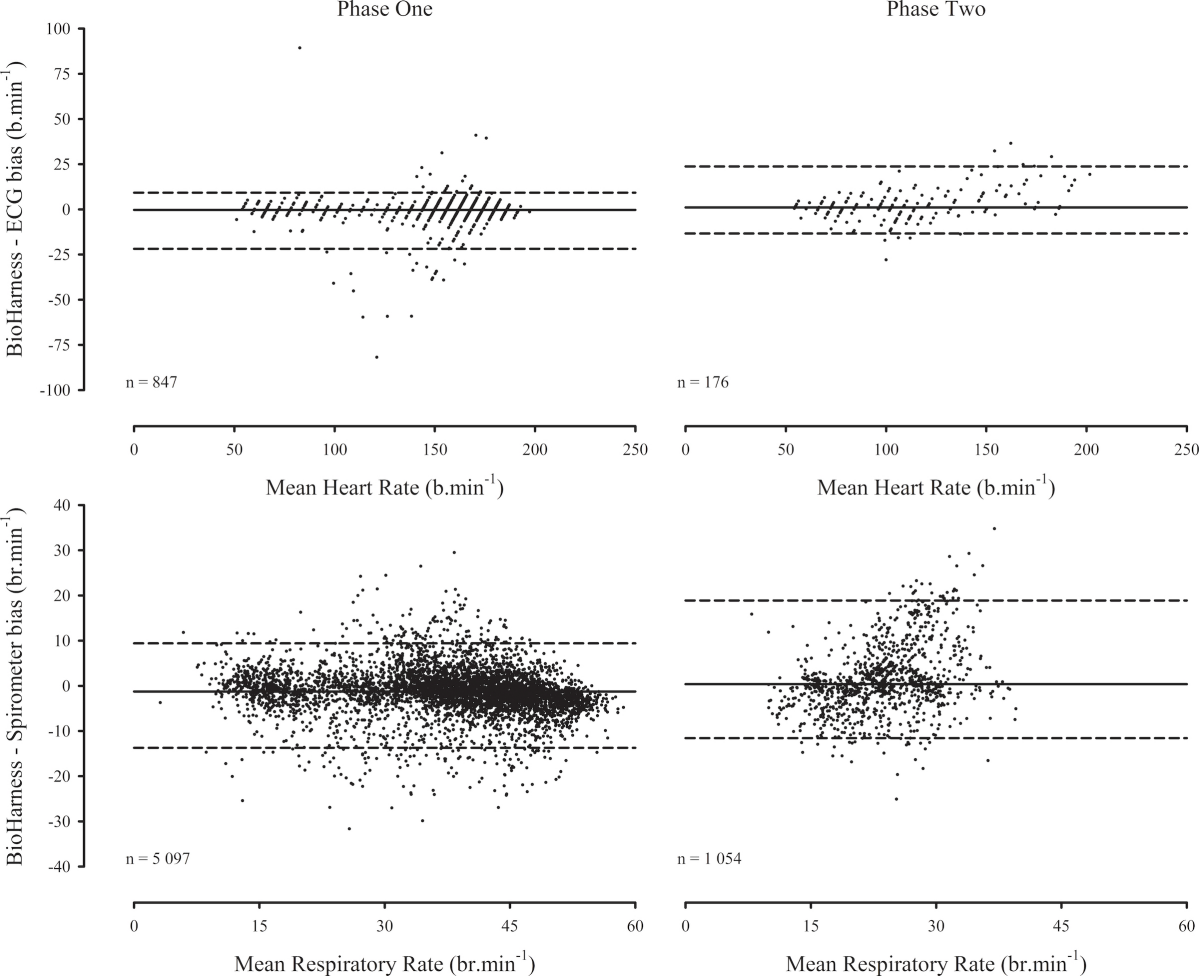
Sensor measurement error as a function of mean measurement magnitude (solid reference lines = mean biases, dashed reference lines = 95% limits of agreement).

BioHarness measurement error was relatively consistent across the measurement ranges, although a degree of heteroscedasticity was apparent among Phase Two respiratory rate measures (Figure 3).

**Table 2:** Biases between BioHarness and reference heart rate (Phase One: participants in sinus rhythm; Phase Two participants with atrial fibrillation)^a^.

			Heart rate		
			Median b∙min^-1^	Bias b∙min^-1^	Bias %
Phase One	Rest	REF	72.00 (18.00)	0.00 (4.45)	0.02 (5.55)
		BH	70.75 (23.38)		
	Transition	REF	108.00 (30.00)	-0.80 (7.20)	-0.65 (7.38)
		BH	97.00 (31.18)		
	Run	REF	162.00 (18.00)	-0.30 (4.60)	-0.20 (2.80)
		BH	163.50 (16.40)		
	Total	REF	162.00 (24.00)	-0.30 (4.53)^b^	-0.20 (2.96)
		BH	160.70 (13.40)		
Phase Two	Rest	REF	84.00 (24.00)	2.10 (4.55)	2.06 (5.77)
		BH	89.10 (29.45)		
	Transition	REF	108.00 (6.00)	1.10 (9.30)	1.67 (8.78)
		BH	91.70 (21.30)		
	Walk	REF	126.00 (40.50)	0.65 (9.25)	0.49 (7.82)
		BH	130.20 (46.58)		
	Cycle	REF	120.00 (60.00)	1.90 (11.50)	1.79 (9.66)
		BH	121.80 (72.50)		
	Sweep	REF	108.00 (27.00)	-3.60 (12.00)	-3.75 (9.70)
		BH	103.10 (20.05)		
	Vacuum	REF	108.00 (30.00)	3.20 (13.76)	3.47 (13.46)
		BH	101.30 (33.34)		
	Total	REF	108.00 (48.00)	1.10 (9.75)^c^	1.23 (8.61)
		BH	106.55 (51.68)		

^a^Table reports median (IQR) reference (REF) and BioHarness (BH) heart rates, absolute (b·min^-1^) and relative (%) biases.

^b^ *P*=.003.

^c^ *P*=.001.

**Table 3:** Biases between BioHarness and reference respiratory rate (Phase One: participants in sinus rhythm; Phase Two participants with atrial fibrillation)^a^.

			Median br∙min^-1^	Bias br∙min^-1 b^	Bias %
Phase One	Rest	REF	17.00 (6.75)	-0.28 (4.00)^tR^	-1.56 (23.42)
		BH	16.15 (5.44)		
	Transition	REF	19.30 (7.33)	-2.20 (5.72)^rR^	-12.17 (29.52)
		BH	17.65 (6.32)		
	Run	REF	41.70 (12.00)	-1.36 (4.58)^rt^	-3.30 (10.65)
		BH	40.80 (10.09)		
	Total	REF	39.90 (15.30)	-1.25 (4.65)^c^	-3.33 (12.01)
		BH	39.02 (13.40)		
Phase Two	Rest	REF	18.50 (6.65)	-0.88 (4.30)^twcsv^	-4.89 (21.77)
		BH	17.29 (4.80)		
	Transition	REF	19.70 (8.30)	-5.73 (5.97)^rwcsv^	-28.02 (23.69)
		BH	14.34 (4.92)		
	Walk	REF	22.30 (5.85)	0.81 (6.34)^rtsv^	3.12 (30.80)
		BH	25.16 (6.35)		
	Cycle	REF	25.00 (7.28)	0.28 (7.65)^rtsv^	1.04 (28.84)
		BH	26.69 (6.94)		
	Sweep	REF	22.00 (6.05)	6.61 (16.35)^rtwcv^	27.22 (77.73)
		BH	31.03 (11.87)		
	Vacuum	REF	19.65 (9.33)	9.42 (10.18)^rtwcs^	43.89 (67.69)
		BH	31.55 (9.72)		
	Total	REF	22.10 (7.18)	0.39 (7.33)^c^	1.56 (31.88)
		BH	24.26 (11.02)		

^a^Table reports median (IQR) reference (REF) and BioHarness (BH) respiratory rates, absolute (br·min-1) and relative (%) biases.

^b^The letters ^r t R^= rest, transition, run; statistically significantly different compared to Phase One rest, transition, Run (*P*<.001 to *P*=.04). The letters ^r t w c s v^ = rest, transition, walk, cycle, sweep, vacuum; statistically significantly different compared to Phase Two rest, transition, walk, cycle, sweep, and vacuum (*P*<.001 to *P*=.02).

^c^Statistically significantly different compared to reference measures (*P*<.001).

### Measurement Reliability

BioHarness and reference heart rate measures were strongly correlated during both phases (Table 4), indicating excellent relative measurement reliability. The SEM, CV, and LoA for heart rate were similar during both phases (Table 4). Small SEM and CV indicate acceptable absolute heart rate measurement reliability during both phases; however, the non-parametrically derived LoA were relatively wide (Figure 3). Asymmetric LoA reflect the aforementioned non-normal measurement error distributions. BioHarness and reference respiratory rate measures were strongly correlated during Phase One, but not Phase Two (Table 4). Phase One respiratory rate SEM and CV were small, but the LoA were relatively wide (Figure 3). The respiratory rate SEM, CV, and LoA were substantially larger during Phase Two (Table 4) and reflect poor absolute measurement reliability. While the magnitude of the Phase Two respiratory rate SEM was comparable to the heart rate SEM during both phases, it represents a larger proportion of the total measurement range and therefore, markedly lower absolute measurement reliability.

**Table 4:** Relative and absolute reliability of BioHarness heart rate and respiratory rate measures^a^.

		Relative		Absolute		
		rho	ICC	SEM, min^-1^	LoA, min^-1^	CV, %
Phase One	HR	.92^b^	.98^b^	5.20	(-21.87, 9.26)	2.24
	RR	.87^b^	.94^b^	2.78	(-13.73, 9.41)	7.94
Phase Two	HR	.97^b^	.98^b^	4.77	(-13.39, 23.79)	4.05
	RR	.43^b^	.55^b^	4.60	(-11.58, 18.91)	16.61

^a^Table reports Spearman’s rank-order correlation coefficient (rho), two-way random effects intraclass correlation coefficient (ICC), standard error of measurement (SEM), non-parametric 95% limits of agreement (LoA), and coefficient of variation (CV) for heart rate (HR) and respiratory rate (RR).

^b^Statistically significant *P*<.001.

### Wireless Data Transmission Reliability

Zero biases were observed between BioHarness, App, and Odin measurements, indicating sensor measurement validity was unaffected by wireless data transmission. Phase One BioHarness, App, and Odin data loss were 4.1%, 0.2%, and 21.3%, respectively. Failure to record data throughout two V̇O_2_peak bouts accounted for all BioHarness data loss. However, these errors did not compromise BioHarness-to-App data transmission. A terminal App crash during one exercise bout accounted for all Phase One App data loss. Outages of the data network hosting the Odin server precluded App-to-Odin data transmission throughout five exercise bouts, and this instability accounted for 15.5% Odin data loss. Unidentified intermittent data capture errors accounted for the remaining Odin data loss (5.9%).

Phase Two BioHarness, App, and Odin data loss were 0.0%, 0.6%, and 1.1%, respectively. Phase Two was unaffected by Odin network stability and data loss occurred as a result of intermittent errors similar to those observed during Phase One.

## Discussion

### Principal Findings

This study evaluated the sensor measurement and wireless data transmission validity of a remote physiological monitoring system among participants in sinus rhythm and AF. Heart and respiratory rates differed systematically from reference measures across a range of exercise intensities and activities, but the magnitudes of these biases were small. Measurement reliability was generally acceptable, and wireless data capture was excellent when all components of the monitoring system were operational. However, instability of the data network hosting the Odin remote monitoring server resulted in substantial data loss during some exercise bouts.

The small magnitudes of heart rate and respiratory rate measurement biases are unlikely to impair interpretation of physiological stress or workload during remote monitoring. As a caveat, larger biases during simulated sweeping and vacuuming may indicate reduced sensor stability and increased movement artefact during activities requiring substantial upper limb movement. Recent evidence suggests conductive fabric sensors embedded in a textile vest are subject to less movement artefact than traditional adhesive ECG electrodes [43]. Thus the BioHarness compression-fit vest may improve sensor measurement validity; however, it was not publicly available during this experiment and could not be assessed.

Heart rate and respiratory rate measurement biases were comparable to some, but not all previous evaluations of similar sensors’ measurement validity. Biases were smaller than those reported for a previous model BioHarness during laboratory- and field-based locomotion [31,32,34] but comparable to those reported during incremental and constant intensity treadmill running [35]. As it is not possible to determine the extent to which iterative hardware and software development contributes to measurement accuracy, caution should be taken when generalizing our results to earlier model devices.

Relative heart rate measurement reliability was excellent across a range of activities and workloads. Correlation coefficients compare favorably with evaluations of previous model BioHarness devices [32,35] and other wearable physiological sensing devices [44-47]. Small SEM, and CV substantially smaller than a previously established criterion for acceptability [45] indicate good absolute heart rate measurement reliability during both phases. Relatively wide LoA are consistent with previous BioHarness evaluations [34,35] and reflect infrequent large measurement errors. While high frequency data are attractive for real-time remote exercise monitoring, the effect of infrequent outlying measurement errors may unnecessarily confound real-time data interpretation. Many wearable physiological sensors support much higher frequency monitoring than is typically provided during center-based supervised exercise. Thus it may be acceptable to sacrifice some temporal resolution in order to increase measurement reliability. Post-processed down-sampling is recommended to account for the temporal instability of respiratory gas exchange data during exercise [48,49], and a similar approach warrants consideration for real-time monitoring of high-frequency physiological data. Aggregating individual data packets, which were transmitted every 30 seconds during this experiment, may overcome the effects of infrequent outlying measurement errors. However, further investigation is required to determine the optimal balance between temporal resolution and measurement reliability.

Respiratory rate measurement reliability was comparable with evaluations of previous BioHarness models [31,35] and other wearable physiological monitors featuring inductive plethysmographs during Phase One [45,47,50] but was notably reduced during Phase Two. This was unexpected, but not without precedent [33,34]. The major methodological discrepancies between phases were the inclusion of upper body activities (simulated sweeping and vacuuming), lower levels of activity intensity, and older aged participants of lower exercise training status during Phase Two. Activities requiring substantial upper limb movement could impair the BioHarness respiratory rate sensor; however, post-hoc sensitivity analyses (not presented) did not support this effect. As low intensity activities are associated with small tidal volumes and thoracic wall displacements [51], it is possible the BioHarness respiratory rate sensor may be confounded during low levels of exercise intensity. Again, however, post-hoc sensitivity analyses did not support this effect. Pulmonary mechanics are impaired among older aged individuals (independent of pathophysiological conditions) and those with respiratory muscle weakness [52,53]. However, data describing pulmonary mechanics were not collected during this study, and the mechanism(s) underlying poor Phase Two respiratory rate measurement reliability remain unknown.

Remote physiological monitoring is contingent on reliable data transmission to a remotely located monitoring station. Data capture was generally excellent throughout this experiment; however, several errors were identified. Unresolved data logging errors precluded data storage on the local BioHarness memory during two exercise bouts; however, remote data transmission was unaffected and all data were successfully transmitted to the remote monitoring server during these errors. While local BioHarness data capture was necessary to assess sensor measurement validity, the middleware platform responds to network instability by temporarily caching all data until a network connection is re-established. Thus local BioHarness data capture would not be required in a production-ready remote monitoring system. The institutional network that hosted the Odin server throughout this study was subject to inconsistent power supply and undisclosed maintenance events. Resulting Odin server outages affected five exercise bouts during three Phase One trials. Relocating Odin to a robust host network will resolve this issue and is an immediate priority for future iterations of the monitoring system. After accounting for host network instability, Odin captured 94.7% and 98.9% of data during Phases One and Two, respectively. Iterative development is required to resolve the remaining App and Odin data capture errors; however, data capture reliability was sufficient for real-time remote monitoring given that a stable App-to-Odin connection was confirmed before beginning exercise.

### Limitations

A potential limitation of this study was the small sample size. However, as the unit of analysis was the number of sensor observations, rather than the number of participants, the design had sufficient statistical power to detect clinically significant biases between BioHarness and reference measures of heart rate and respiratory rate.

As with all studies evaluating physiological sensor validity, these results may be confounded by factors influencing the quality of data from the BioHarness and reference sensors. Positional overlap between ECG (V_1_-V_6_) and BioHarness electrodes may have impaired BioHarness electrode skin contact, particularly among participants with small chest circumferences requiring ECG electrodes to be closely grouped. Interrupted skin contact could explain the occasional presence of large measurement errors apparent in the relatively wide heart rate LoA.

Similarly, the design of the BioHarness respiratory rate sensor dictated that it was typically located above ECG electrodes V_5_ and V_6_. Compression of the respiratory rate sensor against underlying ECG electrodes could impair measurement validity; however, this would be expected to affect both phases and is unlikely to explain the reduced measurement reliability observed during Phase Two.

### Implications

Remote physiological monitoring has numerous potential applications in both clinical and non-clinical settings. Remote monitoring has been identified as an important future development in home-based exCR [26] and may help to bridge the gap between center- and home-based programs for individuals who are unable to attend traditional exCR. Real-time remote physiological monitoring could help home-based exCR participants’ to achieve and adhere to recommended exercise training loads, and this may optimize beneficial exercise-induced physiological adaptations. Moreover, bi-directional communication capability will enable exercise physiologists to provide instantaneous individualized feedback, educational information, and support based on real-time physiological responses. While remote exCR should not replace center-based programs, it may provide a viable alternative for those who are unable or unwilling to attend supervised exCR. Robust trials are now required to determine the efficacy and safety of remotely monitored exCR. Given that center-based exCR is the gold standard treatment in many countries, it seems prudent to compare remotely monitored exCR with center-based programs.

Remote physiological monitoring also has potential applications outside of exCR and could be used to monitor heart rate control in people with AF. Management of patients with AF involves consideration of either a rhythm control approach (attempt to maintain sinus rhythm) or one of rate control, which is often the preferred approach. Reduction of the rapid heart rate in AF increases the diastolic filling periods and left ventricular stroke volume [39]. Current guidelines recommend an individualized approach to AF rate control, using a combination of pharmacological agents such as beta-blockers, calcium channel blockers, and digoxin [39]. However, heart rate control during exercise remains problematic for many patients with AF, even when receiving medications. Guidelines recommend that patients who experience symptoms associated with AF during exercise should be assessed during exercise and have their pharmacological treatment titrated to achieve a physiological chronotropic response and avoid bradycardia [39]. The most common approach for monitoring arrhythmias during everyday life is Holter monitoring (24 hours to 7 days) [39]. This approach is highly regarded and valuable for clinical decision making; however, it is time and resource intensive to monitor data and can be intrusive for patients. Remote monitoring systems such as the one described in this paper have several advantages over traditional Holter monitoring. The conductive textile electrodes embedded into wearable physiological sensors overcome the discomfort associated with adhesive electrodes. Moreover, data from integrated motion sensors could be used to delineate periods of rest and physical activity, and these contextual data may augment interpretation of heart rate control among patients with AF. Finally, embedding automated data collation and processing within remote monitoring servers can eliminate manual data handling and improve the efficiency of data processing and reporting. Collectively these characteristics could assist physicians to assess the effects of pharmacological intervention and titrate AF patients’ medications in order to optimize heart rate control at rest and during exercise. Future research is needed to determine the utility of such remote monitoring in this and other translational contexts.

### Conclusion

The remote monitoring system evaluated in this experiment has sufficient measurement accuracy for quantifying heart rate and respiratory rate among individuals in sinus rhythm and with AF when gold standard clinical sensors are unavailable. Wireless data transmission reliability was generally excellent. Remote physiological monitoring has potential application as an alternate method for delivering exercise-based cardiac rehabilitation and enhancing the management of heart rate control for individuals with atrial fibrillation.

## Abbreviations

AF: atrial fibrillation
C30: constant intensity 30-minute exercise protocol
CHD: coronary heart disease
CR: cardiac rehabilitation
CV: coefficient of variation
ECG: electrocardiogram
exCR: exercise-based cardiac rehabilitation
GPS: global positioning system
I30: intermittent intensity 30 minute exercise protocol
ICC: intraclass correlation coefficient
LOA: limits of agreement
SEM: standard error of measurement
V̇O2peak: peak oxygen consumption
